# The cell-intrinsic circadian clock is dispensable for lateral posterior clock neuron regulation of *Drosophila* rest-activity rhythms

**DOI:** 10.1016/j.nbscr.2025.100124

**Published:** 2025-04-28

**Authors:** Charlene Y.P. Guerrero, Madelyn R. Cusick, Amanda J. Samaras, Natalie S. Shamon, Daniel J. Cavanaugh

**Affiliations:** Department of Biology, Loyola University Chicago, Chicago, IL, United States

**Keywords:** *Drosophila*, Circadian, Clock network, Lateral posterior clock neurons, Locomotor activity

## Abstract

Circadian control of behavior arises from intercommunication among a distributed network of circadian clock neurons in the brain. Single-cell sequencing and brain connectome data support the division of the ∼240 brain clock neurons in *Drosophila* into ∼20 subclusters, and functional studies demonstrate that these populations differentially contribute to behavioral outputs. Here, we have used genetic tools that enable highly selective, cell-specific manipulations to investigate the role of molecular clock function and neuronal activity within the lateral posterior clock neurons (LPNs) in the regulation of rest-activity rhythms. We find that genetic silencing of these neurons, which compromises signaling with downstream neuronal targets, substantially reduces the strength of free-running rest-activity rhythms. In contrast, locomotor activity patterns are robust to CRISPR-mediated disruption of molecular clock cycling within the LPNs. We conclude that the LPNs act as driven oscillators that retain the capacity to transmit circadian information in the absence of cell-intrinsic molecular clocks.

## Highlights

• Elimination of the LPN molecular clock is without effect on *Drosophila* locomotor activity rhythms.
• Genetic silencing of the LPNs degrades locomotor activity rhythm strength.
• Suggests that the LPNs transmit circadian information in a clock-independent manner.

## 1 Introduction

Circadian clock cells in the brain impose temporal organization on behavioral outputs. Clock cells track time of day through the function of a cell-intrinsic molecular clock that acts as a transcriptional-translational feedback loop in which positive elements drive expression of negative elements that subsequently inhibit their own expression. In the fruit fly, *Drosophila melanogaster*, the positive elements are the transcription factors Clock (CLK) and Cycle (CYC), which bind to E-boxes in the promoter regions of the genes encoding the negative elements, *period* (per) and *timeless* (*tim*), to activate transcription. PER and TIM proteins are translated and accumulate as dimers during the day, eventually translocating to the nucleus to repress CLK/CYC-mediated transcription throughout much of the night. PER and TIM are then degraded, allowing another round of transcription to begin, with the entire process taking ∼24 h to complete (Patke et al., 2020).

Molecular circadian clocks are expressed in individual cells, but robust and precisely timed circadian outputs require communication among a network of brain clock cells (Bulthuis et al., 2019; Hastings et al., 2018; Maywood et al., 2011; Schlichting et al., 2019; Yamaguchi et al., 2003). An understanding of the manner that circadian rhythms are established therefore requires a dissection of the network properties through which circadian information generated by clocks in individual cells is coordinated across clock cell populations and transmitted to downstream regions that directly control behavioral outputs. In *Drosophila*, ∼240 brain clock neurons are distributed throughout the dorsal and lateral aspects of the central brain. Initially, these were divided based on anatomical properties into four groups of lateral neurons and three groups of dorsal neurons: the large and small ventrolateral neurons (lLNvs and sLNvs), the dorsolateral neurons (LNds), the lateral-posterior neurons (LPNs), and dorsal neuron groups 1–3 (DN1-3). More recently, single-cell sequencing and brain connectome analyses have suggested that these can be further subdivided into at least 20 discrete clusters (Ma et al., 2021, 2025; Reinhard et al., 2024) that likely serve unique functions in dictating behavioral patterns. For example, the LNds comprise 3 subtypes that make distinct contributions to *Drosophila* locomotor activity and sleep (Brown et al., 2024). The central circadian clock of the mammalian brain, the superchiasmatic nucleus (SCN), can similarly be divided into multiple molecularly distinct subpopulations that together impose circadian regulation on behavioral outputs (Hastings et al., 2018; Morris et al., 2021; Wen et al., 2020; Xu et al., 2021).

Compared to other *Drosophila* clock neuron subsets, the LPNs are relatively uncharacterized. Initial studies suggested that they are particularly sensitive to temperature inputs, as they are one of a few clock neuron populations that preferentially synchronize to temperature oscillations in the presence of conflicting light and temperature entrainment signals (Miyasako et al., 2007; Yoshii et al., 2005, 2010), although they also entrain to light cycles (Shafer et al., 2006). In line with this, recent brain connectome and electrophysiological studies have shown that LPNs are unique among clock neurons in that they receive direct inputs from temperature-sensitive sensory afferent neurons, which makes them strongly activated by heat stimuli (Alpert et al., 2022; Reinhard et al., 2022; Yuan et al., 2024). Given this, it has been proposed that LPNs integrate circadian and temperature information to modify sleep behavior in the face of temperature changes (Alpert et al., 2022; Yuan et al., 2024). The role of LPNs in supporting free-running behavioral rhythms has received less attention, although one study reported that LPN-ablated flies maintained free-running locomotor activity rhythms under constant light and temperature conditions, albeit at substantially reduced rhythm strength (Reinhard et al., 2022). The necessity of the LPN molecular clock for behavioral rhythms has not been studied.

Recently-developed genetic tools, including the split-GAL4 system, have enabled highly-selective, cell-specific manipulations of discrete subsets of *Drosophila* clock neurons, including the LPNs (Alpert et al., 2022; Dionne et al., 2018; Luan et al., 2006; Reinhard et al., 2022; Sekiguchi et al., 2020; Yuan et al., 2024). Using these tools, researchers have begun to probe the individual and combined contributions of the different clock network populations to behavioral rhythmicity. Here, we have applied these tools to either eliminate molecular clock function or to electrically silence LPNs, and assessed the consequences of these manipulations on sleep and locomotor activity. We find that elimination of LPN molecular clock function is without effect on the amount or rhythmicity of sleep and locomotor activity. In contrast, electrical silencing of LPNs subtly alters sleep architecture, reduces anticipation of light-dark transitions, and substantially degrades free-running rest-activity rhythms. These phenotypes demonstrate that LPNs make essential contributions to behavioral rhythms, but that intrinsic molecular clock function within the LPNs is dispensable for these contributions. This suggests that the ability of LPNs to propagate circadian information can be reinforced by clock-controlled inputs from other nodes of the clock network.

## 2 Materials and methods

### 2.1 Fly lines

Flies were maintained in narrow polystyrene vials (Fisher Scientific) and provided a cornmeal-molasses food consisting of (per L): 1.0 L deionized water, 64.7 g yellow cornmeal, 27.1 g dry active granular yeast, 8.0 g 80–100 mesh agar, 90.0 g unsulphured molasses, with 4.4 mL propionic acid and 2.03 g Tegosept to prevent contamination. The R11B03-p65.AD (attP40) (FBti0187770) and R65D05-GAL4.DBD (attP2) (FBti0191712) fly lines were provided by the Bloomington Drosophila Stock Center (BDSC) and combined to create LPN-spGAL4 (Sekiguchi et al., 2020). UAS-*GFP*nls (FBti0012492) and UAS-CD8:GFP (FBti0012685) were provided by the BDSC. UAS-sgRNA-*per* (attP5), UAS-sgRNA-*tim* (attP5), and UAS-sgRNA-*acp9*8AB (attP5) (Delventhal et al., 2019) fly lines were provided by M. Shirasu-Hiza and combined with UAS-Cas9.P2 (attP2) (FBti0166499), provided by the BDSC, to create UAS-tim^CRISPR^, UAS-per^CRISPR^, and UAS-acp^CRISPR^. 10XUAS-frt-eGFP::Kir2.1-stop-frt-mCherry and 10XUAS-frt-mCherry-stop-frt-eGFP::Kir2.1 (attP2) (Watanabe et al., 2017) were provided by D. Anderson. UAS-*sgg*^Y214F^ (a hypomorphic *sgg* allele) (FBti0026626) and UAS-*dbt*^L^ (FBti0202311) were provided by Orie Shafer. All lines were outcrossed 7 times to the Iso31 background.

### 2.2 Locomotor activity monitoring

Flies were entrained to 12:12 light-dark (LD) conditions at 25^o^C prior to behavioral experiments. Following entrainment, individual male flies, aged 3–6 d, were housed in glass tubes containing a 5 % sucrose and 2 % agar food source at one end of the tube. These tubes were loaded into *Drosophila* Activity Monitors (DAM2; Trikinetics Inc.) for locomotor activity monitoring for 4 d in 12:12 LD followed by 11 d in constant darkness (DD), all at 25^o^C. The DAM system infers locomotor activity based on the breaking of an infrared beam that transects the middle of each behavior tube. DAM beam break readings were recorded every minute. To allow for acclimation, data from the first day in LD or DD were excluded from behavioral analyses. We also excluded data from any fly that was determined to have died during the course of behavioral monitoring based on visual inspection of DAM records.

### 2.3 Generation of normalized locomotor activity average day eduction plots

We created normalized eduction plots for the first 3 d in LD or DD following the acclimation day. We first summed DAM beam break data into 30-min bins using the DAMFileScan program (Trikinetics). We then normalized individual fly activity data by dividing the value from each 30 min bin by the mean activity per 30 min across the entire recording period for that fly. We next determined the mean normalized activity for each 30 min bin across a 24-hr day by averaging the value at that timepoint for each of the 3 d of recording. Finally, we averaged these values across all flies of a given genotype.

### 2.4 Morning and evening anticipation analysis

Anticipation data were analyzed using the PHASE program (Persons et al., 2022). 1-min binned DAM beam break data for individual flies were normalized by total daily activity and averaged across three days in LD, beginning at ZT6 on the second day of recording. A Savitzky-Golay filter (order 3, frame length 241) was applied, minimum and maximum points within 180 min preceding lights on (zeitgeber time 0; ZT0) and lights off (ZT12) were identified, and the area under the curve (AUC) of the filtered data between these points was calculated for each fly.

### 2.5 Sleep analysis

We used DAM data to calculate sleep parameters for the first 3 d in LD or DD following the acclimation day. Sleep was defined as 5 or more consecutive minutes without a beam break (Hendricks et al., 2000). We used the SCAMP program (Vecsey et al., 2024) to quantify total sleep duration (with the stdur function), mean sleep bout length (with the smeandur function), and sleep bout number (with the sfreq function). We also used SCAMP to generate average day sleep eduction plots (with the s30 function).

### 2.6 Free-running circadian period and power analysis

Locomotor activity rhythm period and power were calculated for each individual fly from 1-min binned DAM data using chi-square periodogram analysis with ClockLab software (Actimetrics, Wilmette IL). To ensure accurate period assessment, we included data from the 10 d of DAM recordings in DD conditions following the acclimation day. Rhythm power was defined as the amplitude of the periodogram line at the dominant period minus the chi-squared significance line at a significance of p < 0.01. All flies that survived through the entire monitoring period were included in determining the mean locomotor activity rhythm power. Only rhythmic flies (defined as having a chi-square rhythm power ≥100) were included in calculation of mean period.

### 2.7 Immunohistochemistry

Flies used for immunohistochemical analysis were raised in 12:12 LD conditions. ∼7 d old male flies were anesthetized with CO_2_ at lights-on time and transferred to 100 % ethanol for 1 min, then rinsed briefly in phosphate buffered saline with 0.1 % Triton-X (PBST) before dissection in PBST. Harvested brains were fixed in 4 % paraformaldehyde for 20–40 min, blocked for 60 min in 5 % normal donkey serum in PBST (NDST) and incubated for 24 h in primary antibodies diluted in NDST. Primary antibodies were rabbit anti-GFP 1:1000 (Invitrogen A10262), guinea pig anti-PER 1:1000 (UPR 1140; gift of A. Sehgal), and mouse anti-PDF 1:1000 (Developmental Studies Hybridoma Bank PDFC7; generated by J. Blau). Brains were then washed 3 × 15 min in PBST and incubated for 24 h in secondary antibodies diluted in NDST. Secondary antibodies were FITC donkey anti-rabbit 1:1000 (Jackson 711-095-152), Cy3 donkey anti-guinea pig 1:1000 (Jackson 706-165-148) and Cy5 donkey anti-mouse 1:1000 (Jackson 715-175-151). Following incubation in secondary antibody, brains were washed 3 × 15 min in PBST, cleared for 5 min in 50 % glycerol in PBST, and mounted with Vectashield (Vector Labs).

Immunolabeled brains were visualized with a FLUOVIEW 1000 confocal microscope (Olympus), with capture settings held constant across all brains of a given treatment. To quantify the effects of CRISPR expression, we manually counted the number of nuclei in which PER antibody signal was clearly distinguishable from background staining levels in each clock neuron subset (sLNVs, lLNvs, LNds, LPNs, DN1s and DN2s) in one brain hemisphere per fly. We identified sLNv and lLNv based on cell size and presence of PDF immunoreactivity, and identified the other clock cell populations based on stereotypical anatomical position in the brain.

### 2.8 Statistical analysis

We conducted at least three independent behavioral experiments for each condition, and data from individual experiments were pooled for final analysis using GraphPad Prism 10 software. To account for potential experiment-to-experiment variation, we normalized locomotor activity rhythm power values by dividing the raw chi-square power value with the mean value of control flies from a given experiment. Two-way ANOVA (with time and genotype as variables) was used for comparisons of sleep and locomotor activity eduction plots. To assess for differences between genotypes at individual time points in these plots, we used Dunnett's multiple comparisons posthoc test. Asterisks in these plots indicate that the mean value of a given experimental line differed in the same direction from all relevant control lines, even in cases where we only depicted a subset of all groups in the plot. To compare sleep duration, morning and evening anticipation, sleep bout duration and number, and free-running rhythm power, Brown Forsythe and Welch ANOVA with Dunnett's T3 multiple comparisons posthoc tests were used. Asterisks in these plots indicate that a given experimental line was significantly different, with the same effect direction, from all relevant controls. To compare the number of PER-positive cells within the different clock network populations, Mann-Whitney test was used. For all statistical tests, p < 0.05 was considered significant.

## 3 Results

### 3.1 LPN molecular clocks are dispensable for activity and sleep regulation under light-dark conditions

**Fig. 1:**
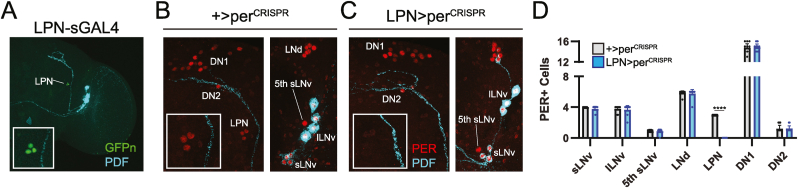
Selective elimination of the LPN molecular clock. (A) Representative, maximum-projection confocal image of a brain from a fly in which LPN-spGAL4 drives expression of nuclear-localized GFP. Green shows GFP antibody staining and cyan shows PDF antibody staining. Inset shows close-up of the GFP-labeled 3 LPNs. (B–C) Representative, maximum-projection confocal images of clock neurons from a control fly brain (left) and a brain in which LPN-spGAL4 is used to drive UAS-per^CRISPR^ expression. Red shows PER antibody staining and cyan shows PDF antibody staining. Insets show the location of the LPN neurons, which lack red PER staining in the LPN-spGAL4>per^CRISPR^ brain (C). (D) Quantification of the number of PER-expressing cells per brain hemisphere in each of the indicated clock network populations from control (gray), and LPN-spGAL4>per^CRISPR^ (blue) brains. Dots represent individual brain hemispheres, and bars show means ± 95 % confidence intervals. n = 9–10 brain hemispheres per group. ∗∗∗∗, p < 0.0001, Mann-Whitney test.

**Fig. S1:**
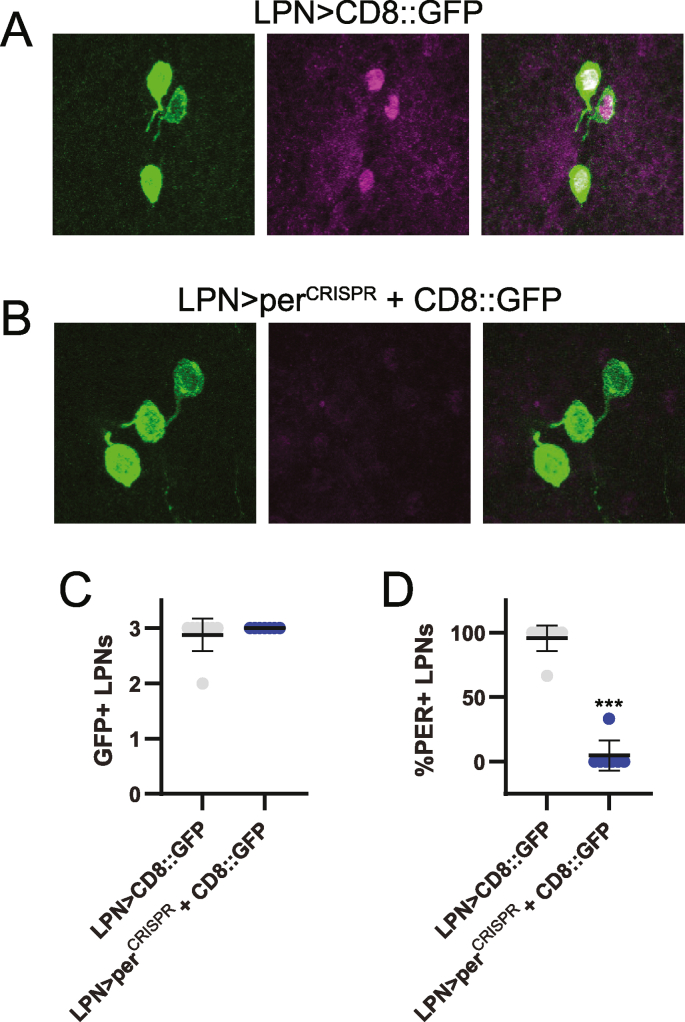
LPNs are retained despite loss of PERIOD staining following CRISPR-mediated elimination of the LPN molecular clock. (A-B) Representative, maximum-projection confocal images of LPN cell bodies from a control fly brain in which LPN-spGAL4 is used to drive UAS-CD8:GFP (A) and an experimental brain in which LPN-spGAL4 is used to drive both UAS-per^CRISPR^ and UAS-CD8:GFP expression (B). Magenta shows PER antibody staining and green shows GFP staining. (C) Quantification of the number of GFP-expressing LPNs per brain hemisphere in control LPN > CD8:GFP (gray), and experimental LPN-spGAL4>per^CRISPR^ + CD8:GFP (blue) brains. Dots represent individual brain hemispheres, and bars show means ± 95 % confidence intervals. (D) Quantification of the percentage of GFP-expressing LPNs per brain hemisphere that are also positive for PER immunoreactivity. n = 7–8 brain hemispheres per group. ∗∗∗, p < 0.001, Mann-Whitney test.

To target the LPNs, we used a recently-developed split-GAL4 line that is selectively expressed in 3 LPNs per brain hemisphere in addition to a handful of cells in the optic lobes (Fig. 1A) (Reinhard et al., 2022; Sekiguchi et al., 2020; Yuan et al., 2024). When we used this split-GAL4 line to drive expression of an inducible CRISPR construct targeting the *period* gene (Delventhal et al., 2019), we observed a complete elimination of PERIOD antibody staining within LPN clock cells, with no effect on other major clock populations (Fig. 1B–D). Analysis of brains from flies co-expressing *period*-targeting CRISPR constructs alongside GAL4-driven CD8:GFP demonstrated that LPN cells were retained at expected numbers despite a lack of PERIOD expression (Fig. S1). Our targeting approach therefore allowed us to determine the consequences of a loss of LPN molecular clocks in the context of an otherwise functional central clock network.

**Fig. 2:**
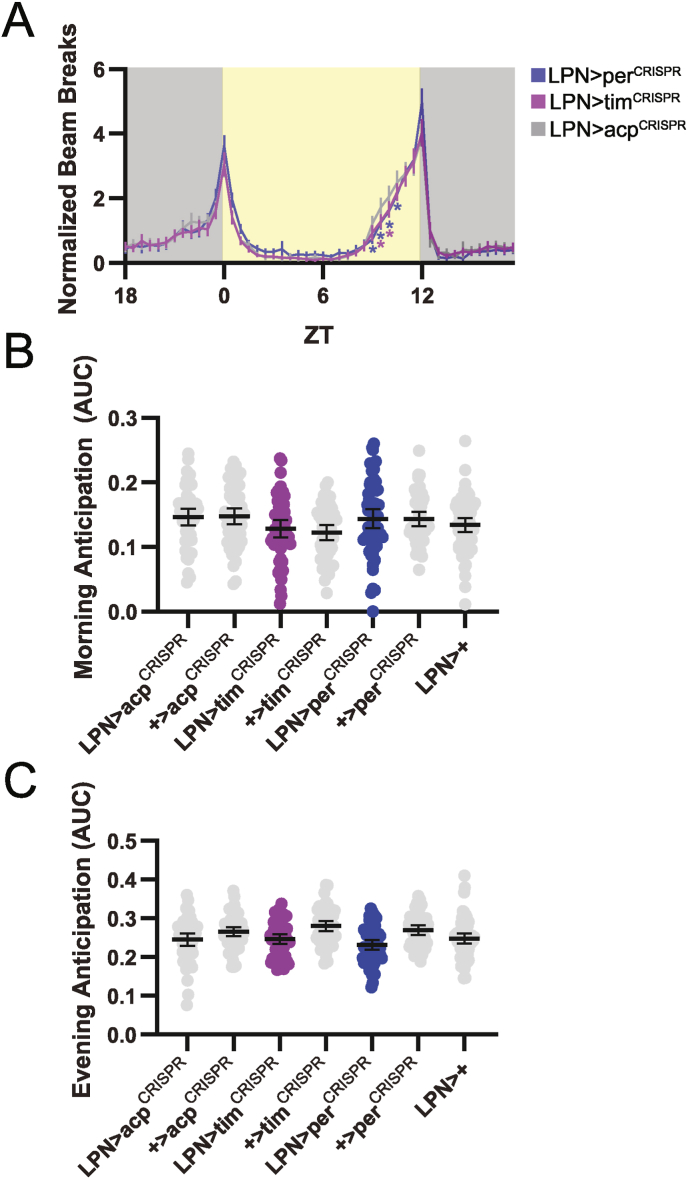
Elimination of the LPN molecular clock does not affect locomotor activity patterns in LD conditions. (A) Eduction plots show locomotor activity across one average LD day for the indicated genotypes. Yellow and gray shading indicates lights on and off, respectively. Lines show mean normalized locomotor activity for each 30 min bin ±95 % confidence intervals. ∗, p < 0.05 compared to all relevant controls, Dunnett's multiple comparisons test following 2-way ANOVA. For simplicity, only one control line is depicted. For a graph that includes all controls, see Fig. S2A. (B–C) Morning and Evening Anticipation are plotted for the indicated genotypes. Dots represent values for individual flies, and lines show means ± 95 % confidence intervals. For all graphs, n = 48–64 flies per genotype. ZT = zeitgeber time.

We began by assessing locomotor activity patterns over 3 days under equinox LD cycles. Normally, flies exhibit bimodal activity peaks in these conditions, with activity ramping up in anticipation of dawn and dusk light transitions, a phenomenon that is dependent on circadian clock function (Allada and Chung, 2010). We found that flies in which LPN-spGAL4 was used to drive expression of *period*- or *timeless*-targeting CRISPR constructs (referred to as LPN > per^CRISPR^ and LPN > tim^CRISPR^ flies) showed LD behavior that was largely indistinguishable from control flies, which either expressed CRISPR constructs targeting *acp98B*, an accessory gland gene that is not involved in circadian clock function (LPN > acp^CRISPR^), or individual UAS or spGAL4 components (+>per^CRISPR^; +>tim^CRISPR^, or LPN>+) (Fig. 2A). We did note a very subtle reduction in normalized activity towards the end of the light period for both LPN > per^CRISPR^ and LPN > tim^CRISPR^ flies, but this did not translate to changes in overall activity or anticipation of the light transitions (Fig. 2B–C).

**Fig. 3:**
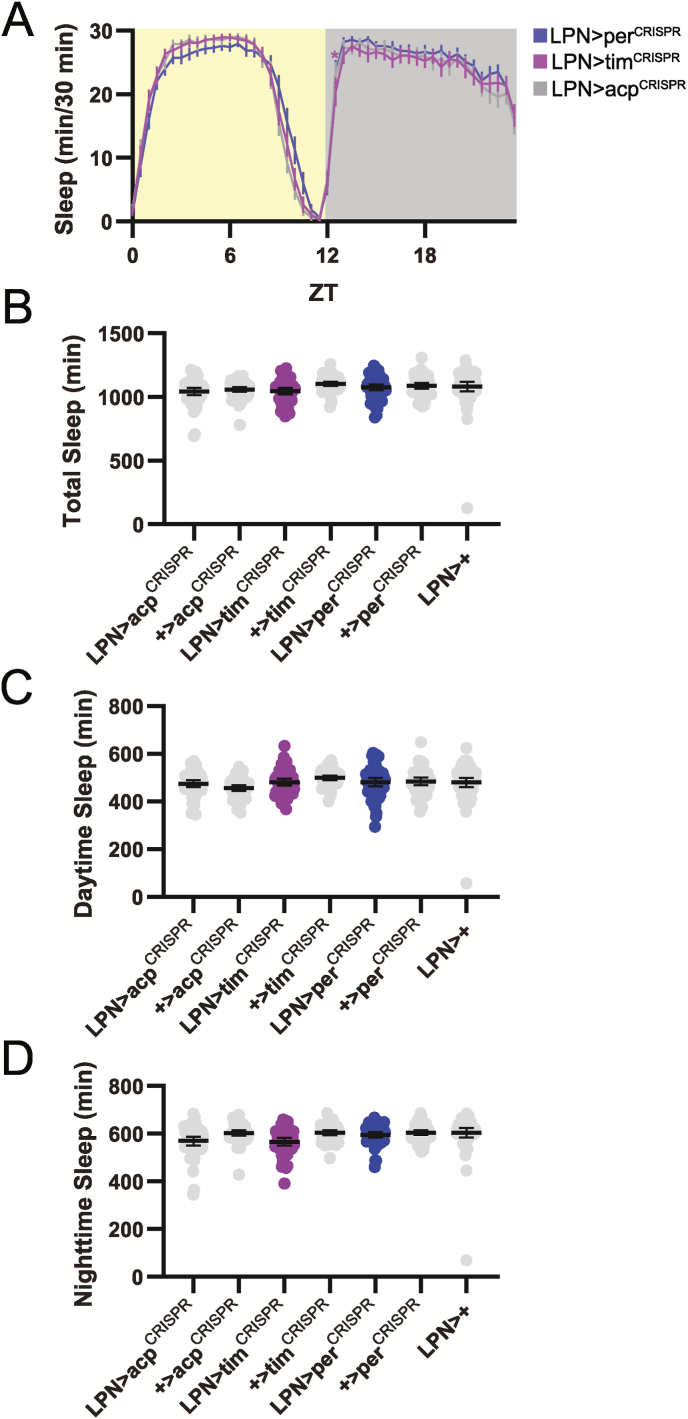
Flies lacking LPN molecular clocks exhibit normal sleep patterns and duration in LD conditions. (A) Eduction plots show sleep across one average LD day for the indicated genotypes. Yellow and gray shading indicates lights on and off, respectively. Lines show mean min of sleep for each 30 min bin ±95 % confidence intervals. ∗, p < 0.05 compared to all relevant controls, Dunnett's multiple comparisons test following 2-way ANOVA. For simplicity, only one control line is depicted. For a graph that includes all controls, see Fig. S2B. (B–D) Average daily sleep duration across the entire 24 h day (B), daytime (lights on) (C), and nighttime (lights off) (D) is plotted for the indicated genotypes. Dots represent values for individual flies, and lines show means ± 95 % confidence intervals. For all graphs, n = 48–64 flies per genotype. ZT = zeitgeber time.

LPN clock ablation was also without effect on sleep behavior under LD conditions. Both LPN > per^CRISPR^ and LPN > tim^CRISPR^ flies exhibited normal sleep patterns, with an extended mid-day siesta and consolidated nighttime sleep separated by brief reductions in sleep during the hours surrounding the dawn and dusk light transitions (Fig. 3A). These flies were also equivalent to control groups in sleep duration (Fig. 3B–D).

### 3.2 LPN molecular clocks are dispensable for activity and sleep regulation under constant environmental conditions

**Fig. 4:**
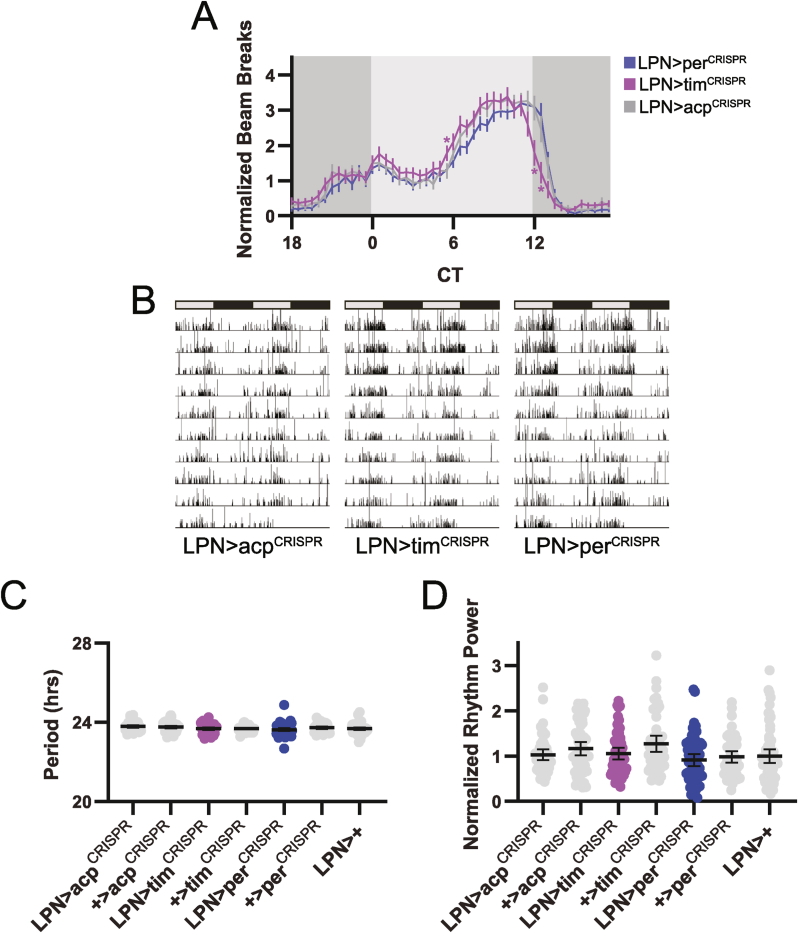
Elimination of the LPN molecular clock does not affect locomotor activity rhythms in DD conditions. (A) Eduction plots show locomotor activity across one average DD day for the indicated genotypes. Light and dark gray shading indicates subjective day and night, respectively. Lines show mean normalized locomotor activity for each 30 min bin ±95 % confidence intervals. ∗, p < 0.05 compared to all relevant controls, Dunnett's multiple comparisons test following 2-way ANOVA. For simplicity, only one control line is depicted. For a graph that includes all controls, see Fig. S2C. (B) Representative double-plotted activity records are shown over 10 DD days for individual flies of the indicated genotypes. Light and dark gray bars indicate subjective day and night, respectively. (C–D) Rhythm period (C) and normalized rhythm power (D) are plotted for the indicated genotypes. Dots represent values for individual flies, and lines show means ± 95 % confidence intervals. For all graphs, n = 48–64 flies per genotype. CT = circadian time.

In the absence of synchronizing environmental cues, organisms maintain free-running behavioral rhythms that proceed with a period dictated by the internal clock. We therefore monitored locomotor activity for 10 d in DD to determine whether LPN clocks are necessary for behavioral patterns under constant environmental conditions. On a group level, flies lacking LPN molecular clocks behaved similarly to controls. Although LPN > tim^CRISPR^ flies exhibited a sharper reduction in activity towards the end of the subjective day, this was not recapitulated in LPN > per^CRISPR^ flies, which were identical to control lines (Fig. 4A). Furthermore, individual LPN > per^CRISPR^ and LPN > tim^CRISPR^ flies had strong activity rhythms that were qualitatively and quantitatively equivalent to controls, as demonstrated by representative activity records (Fig. 4B), and by chi-square periodogram assessment of activity rhythm period (Fig. 4C) and strength (Fig. 4D).

**Fig. 5:**
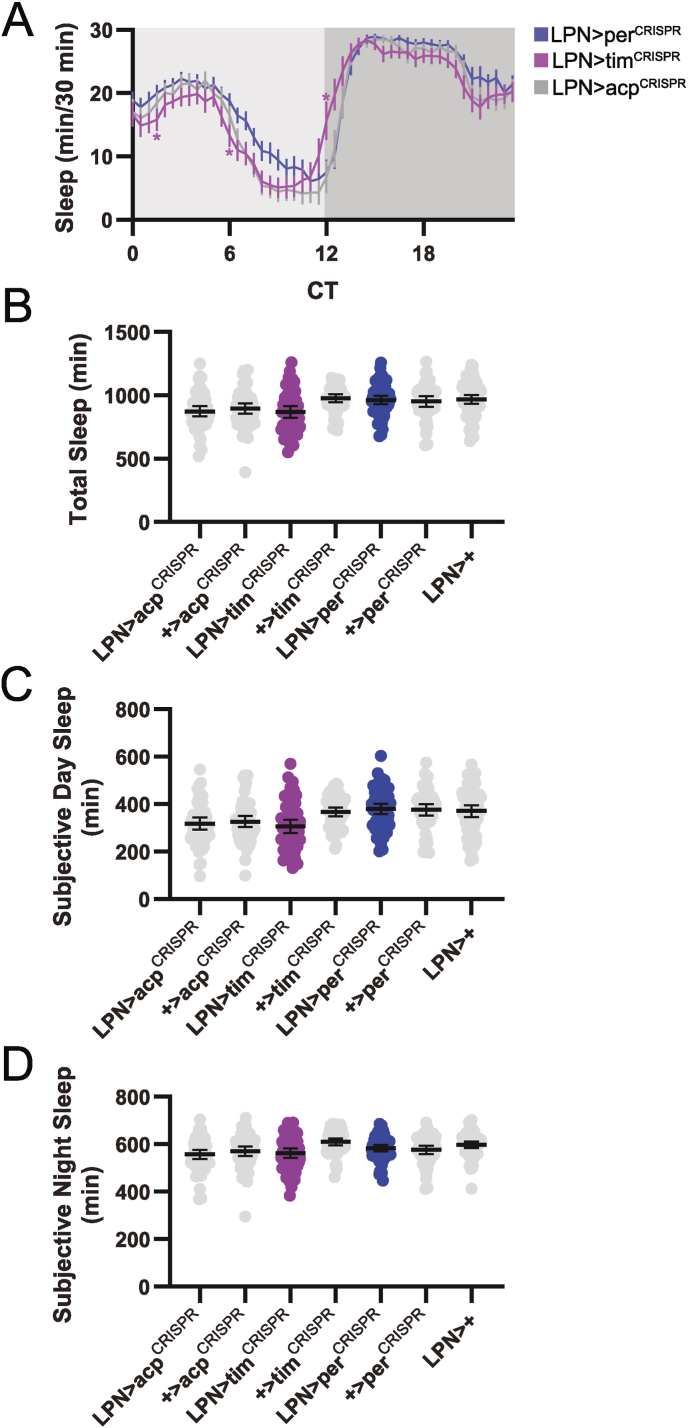
DD sleep behavior is unaltered in flies lacking LPN molecular clocks. (A) Eduction plots show sleep across one average DD day for the indicated genotypes. Light and dark gray shading indicates subjective day and night, respectively. Lines show mean min of sleep for each 30 min bin ±95 % confidence intervals. ∗, p < 0.05 compared to all relevant controls, Dunnett's multiple comparisons test following 2-way ANOVA. For simplicity, only one control line is depicted. For a graph that includes all controls, see Fig. S2D. (B–D) Average daily sleep duration across the entire 24 h day (B), subjective day (C), and subjective night (D) is plotted for the indicated genotypes. Dots represent values for individual flies, and lines show means ± 95 % confidence intervals. For all graphs, n = 48–64 flies per genotype. CT = circadian time.

Concomitant with the lack of effect on locomotor activity, molecular clock abrogation in LPNs did not alter DD sleep behavior. We found no consistent differences in LPN > per^CRISPR^ and LPN > tim^CRISPR^ flies compared to controls in the pattern of sleep across the day (Fig. 5A), nor in the total amount of sleep (Fig. 5B–D). Together with the results discussed above, these findings demonstrate that LPN molecular clocks are dispensable for sleep and locomotor activity regulation under both LD and DD conditions.

### 3.3 Reducing LPN excitability dampens evening anticipation and slightly reduces sleep under LD conditions

**Fig. 6:**
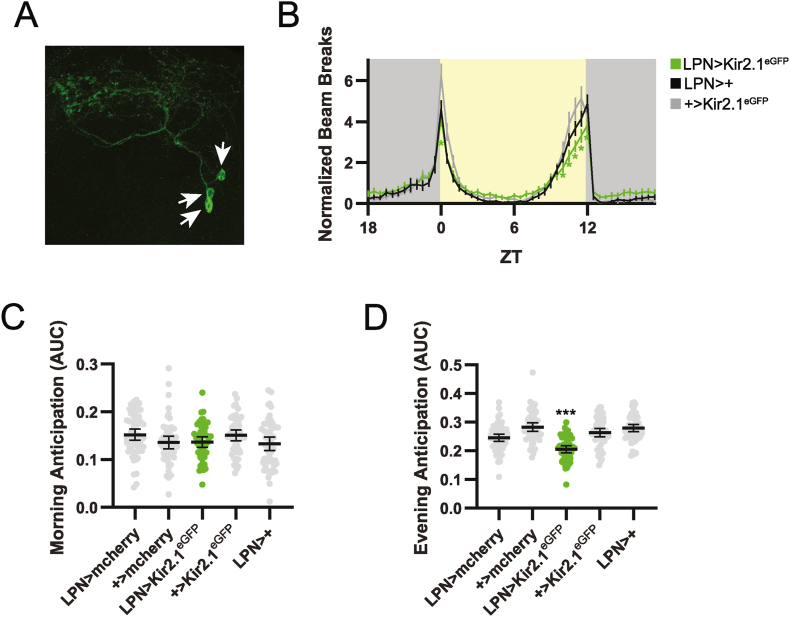
Kir2.1eGFP-induced silencing of LPNs reduces evening anticipation in LD conditions. (A) Representative maximum-projection confocal image of the dorsal region of a brain in which LPN-spGAL4 drives expression of Kir2.1^eGFP^. GFP antibody staining is shown in green. Arrows show 3 eGFP-positive LPN cell bodies. (B) Eduction plots show locomotor activity across one average LD day for the indicated genotypes. Yellow and gray shading indicates lights on and off, respectively. Lines show mean normalized locomotor activity for each 30 min bin ±95 % confidence intervals. ∗, p < 0.05 compared to all relevant controls, Dunnett's multiple comparisons test following 2-way ANOVA. For simplicity, only two control lines are depicted. For a graph that includes all controls, see Fig. S2E. (C–D) Morning and Evening Anticipation are plotted for the indicated genotypes. Dots represent values for individual flies, and lines show means ± 95 % confidence intervals. ∗∗∗, p < 0.001 compared to all relevant controls, Dunnett's T3 multiple comparisons test following Brown-Forsythe and Welch ANOVAs. For all graphs, n = 49–57 flies per genotype. ZT = zeitgeber time.

Expression of *per*- or *tim*-targeting CRISPR constructs eliminates cell-intrinsic molecular clock function within the LPNs but leaves intact their ability to communicate with downstream neuronal targets. To assess whether neuronal signaling emanating from LPNs contributes to behavioral rhythmicity, we monitored locomotor activity and sleep in flies in which we used the GAL4-UAS system to drive expression of an eGFP-labeled Kir2.1 potassium channel (Kir2.1^eGFP^), which hyperpolarizes neurons, rendering them hypoexcitable (Baines et al., 2001; Watanabe et al., 2017). Control flies used LPN-spGAL4 to express a UAS-mcherry construct inserted into the same genomic location as the Kir2.1 construct (LPN > mcherry; see methods) or individual spGAL4 or UAS components (+>Kir2.1^eGFP^ or LPN>+). Under LD conditions, flies expressing Kir2.1^eGFP^ in LPNs (LPN > Kir2.1^eGFP^ flies; Fig. 6A) exhibited reduced normalized activity towards the end of the light period (Fig. 6B), which resulted in a reduction in evening anticipation (Fig. 6D), with no change in morning anticipation (Fig. 6C).

**Fig. 7:**
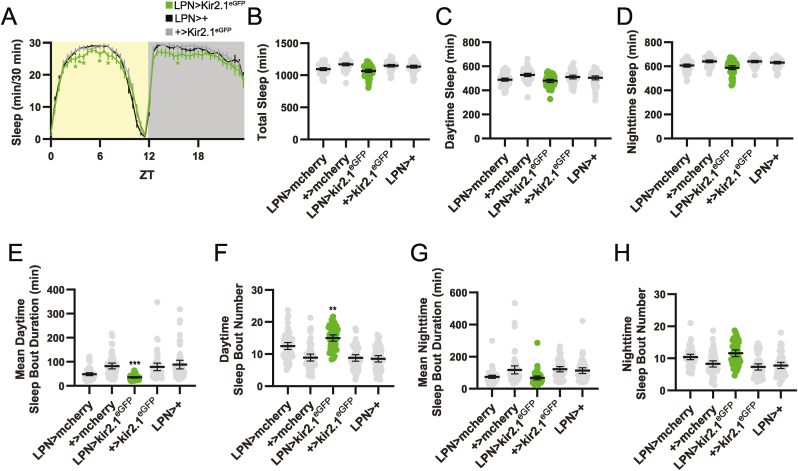
LPN silencing reduces siesta sleep in LD conditions. (A) Eduction plots show sleep across one average LD day for the indicated genotypes. Yellow and gray shading indicates lights on and off, respectively. Lines show mean min of sleep for each 30 min bin ±95 % confidence intervals. ∗, p < 0.05 compared to all relevant controls, Dunnett's multiple comparisons test following 2-way ANOVA. For simplicity, only two control lines are depicted. For a graph that includes all controls, see Fig. S2F. (B–D) Average daily sleep duration across the entire 24 h day (B), daytime (lights on) (C), and nighttime (lights off) (D) is plotted for the indicated genotypes. For B-D, experimental lines are not significantly different from LPN > mcherry controls based on Dunnett's T3 multiple comparisons test following Brown-Forsythe and Welch ANOVAs (for B, p = 0.418; for C, p = 0.942; for D, p = 0.381). (E–F) Mean daytime sleep bout duration (E) and number (F) are plotted for the indicated genotypes. ∗∗∗, p < 0.001; ∗∗p < 0.01 compared to all relevant controls Dunnett's T3 multiple comparisons test following Brown-Forsythe and Welch ANOVAs. (G–H) Mean daytime sleep bout duration (G) and number (H) are plotted for the indicated genotypes. Experimental flies are not significantly different from LPN > mcherry controls based on Dunnett's T3 multiple comparisons test following Brown-Forsythe and Welch ANOVAs (for G, p = 0.872; for H, p = 0.376). For B-H, dots represent values for individual flies, and lines show means ± 95 % confidence intervals. For all graphs, n = 49–57 flies per genotype. ZT = zeitgeber time.

LPN > Kir2.1^eGFP^ flies also had reduced sleep, though this was moderate and time-of-day specific. Pairwise comparisons demonstrated a specific decrease compared to all control lines in sleep in the middle of the daytime siesta (Fig. 7A), though this reduction did not achieve statistical significance when calculated across the entire day (Fig. 7B), or separately for the light or dark periods (Fig. 7C–D). Nevertheless, LPN > Kir2.1^eGFP^ flies displayed a significant decrease in mean daytime sleep bout duration (Fig. 7E) and a concomitant increase in daytime sleep bout number (Fig. 7F), which are indicative of a reduction in daytime sleep consolidation. In contrast, nighttime sleep architecture was not significantly altered compared to all relevant controls (Fig. 7G–H).

### 3.4 Reducing LPN excitability degrades free-running rest-activity rhythm strength

**Fig. 8:**
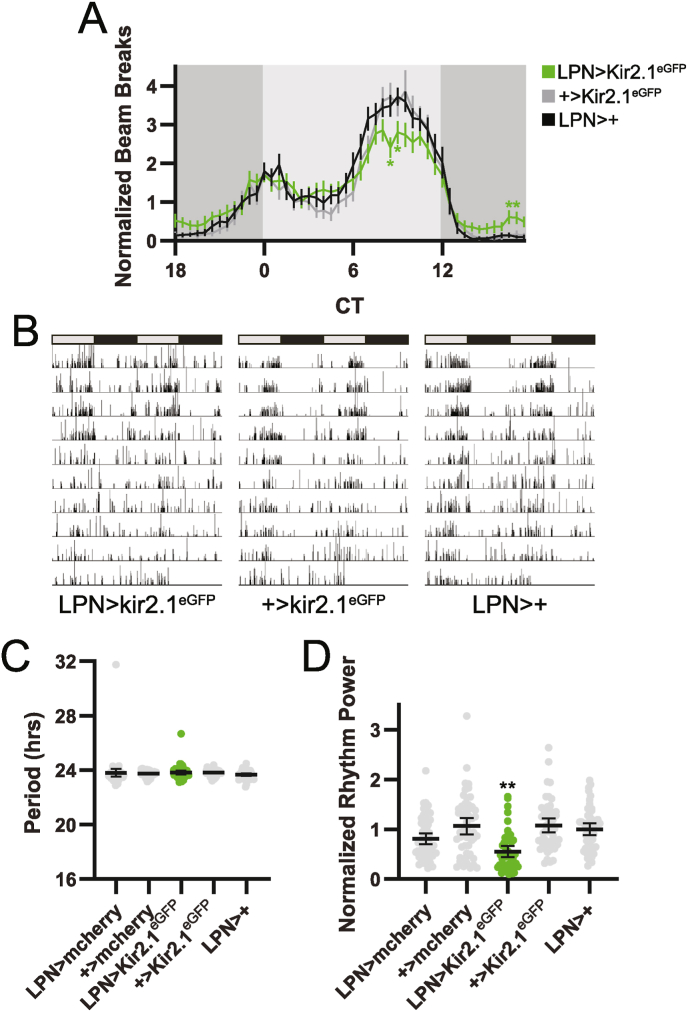
LPN silencing degrades free-running locomotor activity rhythm strength. (A) Eduction plots show locomotor activity across one average DD day for the indicated genotypes. Light and dark gray shading indicates subjective day and night, respectively. Lines show mean normalized locomotor activity for each 30 min bin ±95 % confidence intervals. ∗, p < 0.05 compared to all relevant controls, Dunnett's multiple comparisons test following 2-way ANOVA. For simplicity, only two control lines are depicted. For a graph that includes all controls, see Fig. S2G. (B) Representative double-plotted activity records are shown over 10 DD days for individual flies of the indicated genotypes. Light and dark gray bars indicate subjective day and night, respectively. (C–D) Rhythm period (C) and normalized rhythm power (D) are plotted for the indicated genotypes. Dots represent values for individual flies, and lines show means ± 95 % confidence intervals. ∗∗, p < 0.01 compared to all relevant controls, Dunnett's T3 multiple comparisons test following Brown-Forsythe and Welch ANOVAs. For all graphs, n = 49–57 flies per genotype. CT = circadian time.

In contrast to the lack of effect of molecular clock ablation, electrical silencing of LPNs degraded free-running locomotor activity rhythms under DD conditions. On the group mean level, this manifested as a reduction in normalized locomotor rhythm amplitude, with LPN > Kir2.1^eGFP^ flies displaying a reduction in relative activity towards the end of the subjective day and slight increase in relative activity during the subjective night (Fig. 8A). Individual activity records demonstrated less consolidated periods of activity and rest, with many flies demonstrating an inability to maintain rhythmic activity throughout the 10-d DD period (Fig. 8B). Concomitant with this, mean activity rhythm strength reduced to about 50 % of controls (Fig. 8D). Despite the reduction in rhythm strength, all individual flies retained at least some measure of rhythmicity, and mean period length was unchanged (Fig. 8C). Thus, though LPNs make an important contribution to the partitioning of locomotor activity across the day, neuronal firing in the rest of the central clock network is sufficient to preserve overt rhythmicity.

**Fig. 9:**
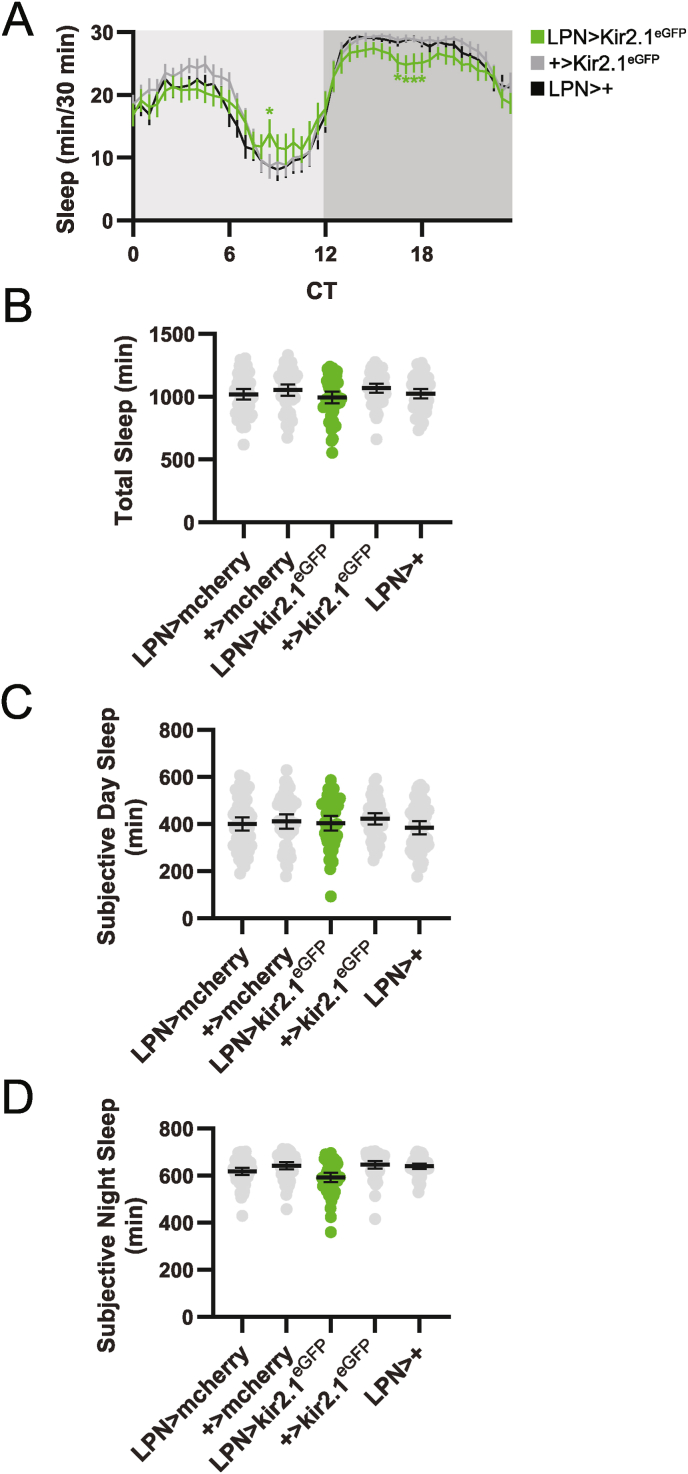
LPN silencing subtly alters DD sleep behavior. (A) Eduction plots show sleep across one average DD day for the indicated genotypes. Light and dark gray shading indicates subjective day and night, respectively. Lines show mean min of sleep for each 30 min bin ±95 % confidence intervals. ∗, p < 0.05 compared to all relevant controls, Dunnett's multiple comparisons test following 2-way ANOVA. For simplicity, only one control line is depicted. For a graph that includes all controls, see Fig. S2H. (B–D) Average daily sleep duration across the entire 24 h day (B), subjective day (C), and subjective night (D) is plotted for the indicated genotypes. Dots represent values for individual flies, and lines show means ± 95 % confidence intervals. For all graphs, n = 49–57 flies per genotype. CT = circadian time.

As was the case in LD, electrical silencing of LPNs slightly reduced DD sleep in a time-of-day dependent manner. In this case, the reduction was specific to the middle of the subjective night (Fig. 9A), but did not reach statistical significance when quantified across the entire day (Fig. 9B), the subjective day (Fig. 9C) or the subjective night (Fig. 9D).

## 4 Discussion

A major focus of chronobiological research is to determine the cellular and circuit-level mechanisms through which circadian regulation is imposed upon behavioral outputs. Pioneering studies in *Drosophila* employing cell-specific manipulations proposed a hierarchical dual-oscillator model of the central clock network in which sLNv and LNd neurons served as ‘morning’ and ‘evening’ cells that drove stereotypical anticipatory increases in activity prior to the dawn and dusk lighting transitions (Grima et al., 2004; Stoleru et al., 2004). These clock network populations were also identified as prominent regulators of free-running activity rhythms under constant light conditions (Picot et al., 2007; Renn et al., 1999). As tools for cell-specific targeting have been refined to allow for investigations of other clock network populations, the dual oscillator model has gradually been supplanted by the idea that clock network hierarchies are flexible depending on factors such as time of day, temperature and lighting conditions, and that, despite important and essential contributions from sLNv and LNd cells, coherent behavioral rhythms require the combined contribution of multiple clock neuron populations (Bulthuis et al., 2019; Chatterjee et al., 2018; Saurabh et al., 2024; Schlichting et al., 2019; Yao et al., 2016; Yao and Shafer, 2014). This may reflect the extensive, reciprocal synaptic and peptidergic connections between the different clock cell populations (Reinhard et al., 2024; Shafer et al., 2022), which could allow for integration and feedback between clock network cells to sculpt output signals emanating from the clock network.

Consistent with this expanding model, our results demonstrate an important role for LPN clock neuron firing in maintaining robust locomotor activity rhythms. It is possible that the degradation in behavioral rhythmicity associated with LPN silencing reflects an impact of loss of LPN function in communication and synchronization within the clock network. Connectomic studies have identified synaptic connections between LPNs and multiple other clock cell populations, including DN1ps, DN3s and LNds (Reinhard et al., 2022, 2024), and recent calcium imaging experiments confirmed direct functional connectivity between LPNs and LNds (Yuan et al., 2024). In addition to synaptic signaling, LPNs release several neuropeptides that could act in a paracrine fashion to target additional clock groups, including sLNvs (Ma et al., 2021; Reinhard et al., 2022, 2024). The LPNs are thus situated to contribute to inter-clock network synchronization and coherence. Immunohistochemical assessment of molecular clock cycling in the face of LPN silencing would provide a formal test of whether clock network synchrony is affected when LPN signaling is eliminated. The impact of LPN silencing on behavioral rhythmicity could also occur due to disruption of communication between LPNs and downstream output neurons. LPNs make extensive contacts with neurons outside the clock network, including several populations of descending neurons that could alter locomotor activity through connections with motor circuits in the ventral nerve cord (Reinhard et al., 2022, 2024).

We emphasize that LPN silencing dampened but did not eliminate locomotor activity rhythms. Individual LPN > Kir2.1^eGFP^ flies retained some degree of behavioral rhythmicity, and group averages demonstrate substantial daily oscillations in locomotor activity (Fig. 8A). This stands in contrast to the more drastic impact of silencing other clock cell populations (Bulthuis et al., 2019; Depetris-Chauvin et al., 2011; Guo et al., 2014; Nitabach et al., 2002; Saurabh et al., 2024), and suggests that LPNs refine locomotor activity rhythms but are not absolutely required for their presence. One caveat is that although we were able to visualize ectopic Kir2.1 expression in LPN neurons through immunohistochemical imaging of the eGFP tag attached to the channel (Fig. 6A), we did not electrophysiologically confirm the effect of Kir2.1^eGFP^ expression on LPN firing. Interestingly, we found that in a few brain hemispheres, we could only detect 2 Kir2.1^eGFP^-labeled LPNs, and in other hemispheres, we observed that 1 of the 3 LPNs exhibited comparatively weaker Kir2.1^eGFP^ expression. It is thus possible that LPN silencing may not be complete across all LPNs in all brains, potentially permitting residual contributions to rhythmicity. However, our findings of a ∼50 % reduction in rhythm strength in LPN > Kir2.1^eGFP^ flies are of a similar magnitude to those observed by Reinhard et al. following expression of the cell death gene *hid* in LPNs, which was confirmed to completely ablate LPNs and therefore to abolish LPN signaling (Reinhard et al., 2022).

We also note that the LPN-spGAL4 line we have used drives expression in a number of intrinsic optic lobe neurons in addition to marking the LPNs (Reinhard et al., 2024; Sekiguchi et al., 2020; Yuan et al., 2024). These optic lobe neurons do not express clock components, and therefore would not be affected by our CRISPR manipulations, but they are likely inhibited following LPN-spGAL4-mediated expression of Kir2.1^eGFP^. Thus, we cannot exclude the possibility that the behavioral phenotypes we observed in LPN > Kir2.1^eGFP^ flies derive in part from inhibition of optic lobe neurons. We believe that it is unlikely that optic lobe neuron silencing contributes to the degradation of free-running activity rhythms that occurs in flies in which LPN-spGAL4 drives Kir2.1^eGFP^ expression, given that these rhythms persist unaffected in visually blind flies. However, other behavioral phenomena such as morning and evening anticipation, which are altered by the presence of light cues, could be impacted by compromised visual processing in the optic lobe (Helfrich-Förster et al., 2001; Schlichting, 2020). To unequivocally rule out a contribution of these optic lobe neurons would require selective LPN manipulation. This has previously been achieved through a complicated genetic approach involving simultaneous use of multiple intersectional targeting strategies (Yuan et al., 2024). Interestingly, this approach demonstrated that the acute sleep-promoting effect of LPN-spGAL4 activation persisted in the absence of concurrent optic lobe neuron stimulation.

**Fig. S3:**
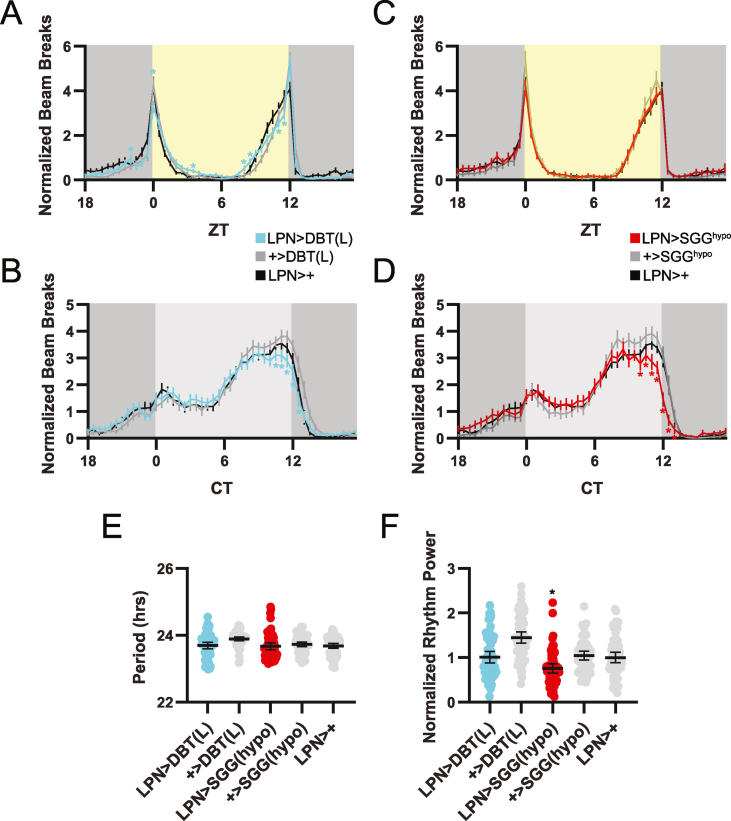
Slowing down the LPN molecular clock reduces locomotor activity rhythm coherence. (A-D) Eduction plots show locomotor activity across one average LD (A and C) or DD (B and D) day for the indicated genotypes. For LD plots, yellow and gray shading indicates lights on and off, respectively. For DD plots, light and dark gray shading indicates subjective day and night, respectively. Lines show mean normalized activity for each 30 min bin ±95 % confidence intervals. ∗, p < 0.05 compared to all relevant controls, Dunnett's multiple comparisons test following 2-way ANOVA. (E-F) DD rhythm period (E) and normalized rhythm power (F) are plotted for the indicated genotypes. Dots represent values for individual flies, and lines show means ± 95 % confidence intervals. ∗, p < 0.05 compared to all relevant controls, Dunnett's T3 multiple comparisons test following Brown-Forsythe and Welch ANOVAs. For all graphs, n = 57–60 flies per genotype. ZT = zeitgeber time; CT = circadian time.

In contrast to the significant reduction in rhythm strength associated with LPN silencing, elimination of molecular clock function within these cells was essentially without effect on the timing or rhythmicity of locomotor activity and sleep. We hypothesize that this is because neuronal inputs from other central clock network populations can drive molecular and physiological cycles in the LPNs in the absence of clock function, allowing LPNs to transmit circadian information even in the absence of an intrinsic time-keeping mechanism. However, this does not preclude a functional contribution of the LPN molecular clock. In fact, we found that LPN-specific expression of a hypomorphic allele of shaggy (SGG) kinase that speeds up molecular clock oscillations (Martinek et al., 2001; Yao and Shafer, 2014) significantly alters behavioral activity patterns, phase-advancing the downslope of the subjective evening activity peak (Fig. S3D) and subtly reducing DD locomotor activity rhythm strength to ∼75 % of control values (Fig. S3E). Thus, though it may be rendered dispensable by a redundant contribution from clock network inputs, the LPN molecular clock still appears to modulate LPN function such that rhythm coherence is reduced when it is forced into misalignment with molecular clocks in other nodes of the clock network.

In addition to modifying activity patterns, LPNs have also been found to regulate sleep, although there are conflicting findings on whether LPNs promote or inhibit sleep. It was initially reported that optogenetic activation of neurons that express the neuropeptide Ast-A, which is enriched in LPNs, increased daytime sleep (Ni et al., 2019). Consistent with these findings, optogenetic stimulation of LPNs had a very strong sleep-promoting effect, while manipulations that prevented LPN neurotransmitter release drastically decreased total sleep amount (Yuan et al., 2024). Together, these studies suggest a sleep-promoting role for LPNs. In contrast, LPN ablation was found to cause a minor increase in sleep duration, which would indicate a sleep-inhibitory role for these cells. It is not clear what may underlie the differential effects found in these various studies. Notably, the latter two studies used the same LPN-spGAL4 line that we used in the current study and came to conflicting conclusions about whether LPNs promote or inhibit sleep. It is important to note that connectomic and gene expression studies indicate that the LPNs constitute two discrete neuron populations (Ma et al., 2025; Reinhard et al., 2022, 2024). It has been proposed that these may differentially regulate sleep (Ma et al., 2025; Reinhard et al., 2022), such that the extent to which a given manipulation affects one or both of these LPN subgroups may bias towards sleep promotion or inhibition, though this conjecture lacks formal confirmation, which would require selective manipulation of the different LPN subgroups. The direction and magnitude of the effect may also depend on whether the manipulation is acute, as in the case of optogenetic stimulation, or chronic, as in the case of cell ablation. Thus, though our results showing that Kir2.1^eGFP^ expression depresses sleep in a time-of-day dependent manner support the idea that LPNs generally promote sleep, they do not rule out a more nuanced role depending on environmental and experimental factors.

Our behavioral assessments were all conducted under constant temperature conditions, but several reports indicate that LPNs may be especially sensitive to temperature cues. For example, the LPN molecular clock readily entrains to temperature cycles (Miyasako et al., 2007; Yoshii et al., 2005) and LPNs are acutely excited by heating (Alpert et al., 2022). Studies also indicate that LPNs contribute both to acute effects of temperature on sleep, and to the ability of flies to synchronize locomotor activity rhythms to temperature cycles. Whereas flies normally increase daytime sleep in response to exposure to temperature increases, this is largely attenuated when tetanus toxin is expressed in LPNs, which prevents neurotransmitter release from these cells (Alpert et al., 2022; Yuan et al., 2024). Furthermore, LPN ablation delays behavioral re-entrainment to shifted temperature cycles (Reinhard et al., 2022), although temperature entrainment persists following LPN-spGAL4-driven expression of a dominant-negative CYC construct that should prevent molecular clock cycling (Lorber et al., 2022). In light of these findings, it will be of importance to conduct additional studies in which temperature is modulated across the day to further investigate the extent to which LPNs, and the molecular clocks expressed therein, function either to mediate temperature entrainment or to behavioral responses to temperature change. Such studies would increase our understanding of the manner through which the *Drosophila* circadian system integrates circadian and temperature information to allow the central clock network to adapt behavior in an ecologically effective manner.

## CRediT authorship contribution statement

**Charlene Y.P. Guerrero:** Writing – review & editing, Validation, Investigation, Formal analysis, Data curation. **Madelyn R. Cusick:** Writing – review & editing, Validation, Investigation, Formal analysis, Data curation. **Amanda J. Samaras:** Writing – review & editing, Visualization, Investigation, Formal analysis, Data curation. **Natalie S. Shamon:** Writing – review & editing, Visualization, Investigation, Formal analysis, Data curation. **Daniel J. Cavanaugh:** Writing – original draft, Visualization, Supervision, Investigation, Funding acquisition, Formal analysis, Conceptualization.

## Funding

This study was funded by the 10.13039/100000001National Science Foundation, 10.13039/100000154Division of Integrative Organismal Systems, CAREER Award 1942167 to D.J.C. The funder played no role in study design, data collection, analysis and interpretation of data, or the writing of this manuscript.

## Declaration of competing interest

None.

## Figures and Tables

**Fig. S2:**
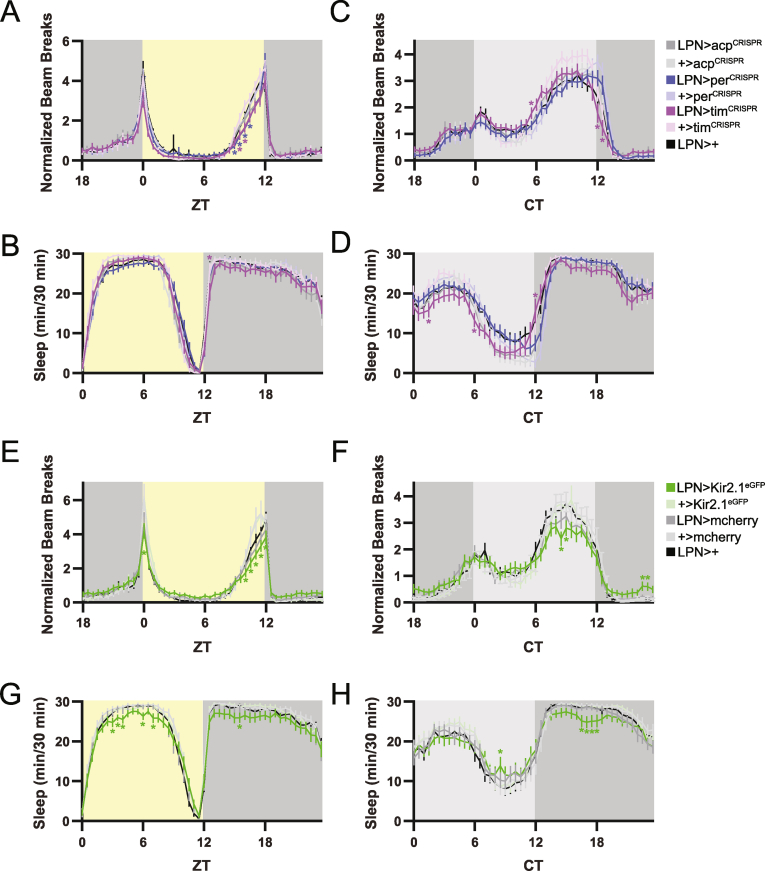
Activity and sleep eduction plots including all control lines. Eduction plots show locomotor activity (A, C, E, G) or sleep (B, D, F, H) across one average LD (A, B, E, F) or DD (C, D, G, H) day for the indicated genotypes. For LD plots, yellow and gray shading indicates lights on and off, respectively. For DD plots, light and dark gray shading indicates subjective day and night, respectively. All lines show mean min of sleep or normalized beam breaks for each 30 min bin ±95 % confidence intervals. ∗, p < 0.05 compared to all relevant controls, Dunnett's multiple comparisons test following 2-way ANOVA. ZT = zeitgeber time; CT = circadian time.

## Data Availability

Data will be made available on request.
